# Carboplatin-loaded surface modified-PLGA nanoparticles confer sustained inhibitory effect against retinoblastoma cell in vitro

**DOI:** 10.5935/0004-2749.20220075

**Published:** 2025-08-22

**Authors:** Hua Zhuang, Ya-Nan Xu, Hong-Hua Zheng, Yu-Rong Huan, Ning-Xuan Zheng, Lin Lin, Wu-Zhen Zhang, Wei Xu

**Affiliations:** 1 Xianyou Maternity and Childcare Hospital, Putian, 351200, Fujian Province, China; 2 Fujian Medical University, Fujian Province, China; 3 Aier School of Ophthalmology, Central South University, Changsha, 410083, Hunan Province, China; 4 Fuzhou Aier Eye Hospital, China; 5 Fujian Center for Disease Control and Prevention, Fu Zhou 350000, Fujian Province, China; 6 Women and Children’s Hospital Affiliated to Xiamen University, Xiamen, 361000, Fujian Province, China; 7 The 1^st^ affiliated Hospital of Fujian Medical University, Fuzhou, Fujian Province, China

**Keywords:** Carboplatin, Alginate, Retinoblastoma, Nanoparticle, Carboplatina, Alginato, Nanopartícula, Retinoblastoma

## Abstract

**Purpose:**

To investigate the antiproliferative effect of carboplatin-loaded surface-modified poly(lactide-*co*- glycolide) on retinoblastoma cells.

**Methods:**

Carboplatinloaded poly(lactide-*co*-glycolide) with or without sodium alginate surface modification was prepared using sodium alginate-poly(lactide-*co*-glycolide) and poly(lactide-*co*-glycolide). The zeta potential and carboplatin release behavior were investigated. The cellular uptake of the released drug was observed in the retinoblastoma cell line Y79. The inhibitory effect of carboplatin-loaded nanoparticles against the Y79 cell line was evaluated using methyl thiazolyl tetrazolium assay and western blot. Native carboplatin and void nanoparticles without carboplatin loading were used as controls.

**Results:**

The zeta potential was -(26.1 ± 3.1) mV for carboplatin-loaded poly(lactide-*co*-glycolide) and-(43.1 ± 8.1) mV for carboplatin-loaded sodium alginate-poly(lactide-*co*-glycolide). The burst release percentages of carboplatin-loaded poly(lactide-*co*-glycolide) and sodium alginate-poly(lactide-*co*-glycolide) were (40.0% ± 8.2%) and (18.9% ± 4.3%) at 24 hours, respectively. A significant difference was identified regarding drug release between carboplatin-loaded sodium alginate-poly(lactide-*co*-glycolide) and carboplatin-loaded poly(lactide-*co*-glycolide). Fluorescence detection revealed that intense uptake of carboplatin into the cytoplasm of the Y79 cell line that was exposed to carboplatin-loaded sodium alginate-poly(lactide-*co*-glycolide). Carboplatin-loaded poly(lactide-*co*-glycolide) or sodium alginate-poly(lactide-*co*-glycolide) exposure inhibited proliferating cell nuclear antigen expression in Y79 cells on day 3. Extension of exposure to day 5 revealed that the sodium alginate-poly(lactide-*co*-glycolide) surface modification was superior to that of poly(lactide-*co*-glycolide) in terms of proliferating cell nuclear antigen inhibition. The cell viability test using methyl thiazolyl tetrazolium revealed a similar inhibitory effect. Furthermore, the carboplatin-loaded nanoparticles of lower concentration inhibited cell viability more strongly than native carboplatin of higher concentration in methyl thiazolyl tetrazolium assay.

**Conclusions:**

Carboplatin-loaded sodium alginate-poly(lactide-*co*-glycolide) inhibited retinoblastoma cell proliferation with superior effect as compared with poly(lactide-*co*-glycolide) and native carboplatin. Sodium alginate surface modification offers a potential strategy for the sustained carboplatin release system.

## Abstract (pt)

**Objetivo:**

Investigar o efeito antiproliferativo de poli (lactídeo-coglicolídeo) com superfície modificada carregada com carboplatina contra células de retinoblastoma.

**Métodos:**

Preparou-se poli (lactídeo-co-glicolídeo) carregado com carboplatina com ou sem alginato de sódio para modifição da superfície, poli com alginato de sódio (lactídeo-co-glicolídeo) e poli (lactídeo-co-glicolídeo). O potencial zeta e o comportamento de liberação de carboplatina foram investigados. A captação celular do fármaco liberado foi observada na linha celular de retinoblastoma Y79. O efeito inibitório das nanopartículas carregadas com carboplatina contra a linha celular Y79 foi avaliado através do ensaio de metiltiazol tetrazólio e Western-blot. Carboplatina nativa e nanopartículas vazias sem carga de carboplatina serviram como controles.

**Resultados:**

O potencial zeta de poli carregado com carboplatina (lactídeo-co-glicolídeo) foi - (26,1 ± 3,1) mV versus - (43,1 ± 8,1) mV em poli com alginato de sódio carregado com carboplatina (lactídeo-co-glicolídeo). A percentagem de libertação de explosão de poli carregado com carboplatina (lactídeo-co-glicolídeo) e poli com alginato de sódio (lactídeo-co-glicolídeo) foram (40,0 ± 8,2)% e (18,9 ± 4,3)% às 24 horas, respectivamente. Uma diferença significativa foi identificada em relação à liberação de fármaco entre poli com alginato de sódio carregado com carboplatina (lactídeo-co-glicolídeo) e poli carregado com carboplatina (lactídeo-co-glicolídeo). A detecção de fluorescência revelou que a carboplatina foi assimilada intensamente no citoplasma da linha celular Y79 que foi exposta ao poli com alginato de sódio carregado com carboplatina (lactídeo-co-glicolídeo). A exposição de poli carregada com carboplatina (lactídeo-co-glicolídeo) ou poli com alginato de sódio (lactídeo-co-glicolídeo) inibiu a expressão de antígeno nuclear de proliferação celular em células Y79 no 3º dia. A extensão da exposição no 5º dia revelou que poli com alginato de sódio (lactídeo-co-glicolídeo) para modificação da superfície foi superior a poli (lactídeo-co-glicolídeo) em termos de inibição do antígeno nuclear de proliferação celular. O teste de viabilidade celular via metiltiazol tetrazólio mostrou um efeito inibitório semelhante. Além disso, as nanopartículas carregadas com carboplatina de concentração mais baixa inibiram a viabilidade celular mais fortemente em comparação com a carboplatina nativa de concentração mais alta no ensaio de metiltiazol tetrazólio.

**Conclusões:**

Poli com alginato de sódio carregado com carboplatina (lactídeo-co-glicolídeo) inibiu a proliferação de células de retinoblastoma com efeito superior em contraste com poli (lactídeo-co-glicolídeo) e carboplatina nativa. O alginato de sódio para modificação da superfície oferece uma estratégia potencial para o sistema de liberação de carboplatina sustentada.

## INTRODUCTION

Retinoblastoma (RB) is a most common pediatric cancer of the eye that compromises the vision and endangers the lives of children. It is caused by a mutation of both alleles of the RB tumor suppressor gene (RB1), and the protein product of RB1 gene regulates a cellular antiproliferative Rb pathway, which when deregulated leads to a series of malignancies^(1)^. Children with RB require treatments, including focal treatment (cryotherapy^(2)^ and laser), chemotherapy (intra-arterial chemotherapy), radiotherapy (external beam radiotherapy and episcleral plaque radiotherapy), and even enucleation^(3,4)^. Among these treatments, intra-arterial chemotherapy can be effective for vitreous disease and causes minimal retinal toxicity^(5)^. However, ophthalmic artery chemotherapy requires a team of specialists and resources that are not available in all RB medical centers. Moreover, for many centers, impermanent efficacy and toxicity^(6)^ remain unsolved problems^(7)^. Other serious side effects of these local treatments include cataract, facial deformities, radiation retinopathy, and even a potential risk of secondary tumors^(8)^. In addition, systemic chemotherapy with cell cycle-blocking medicine is widely used in the treatment of RB. However, clinical use of chemotherapy is also limited by rapid blood clearance, vitreous seeding^(9)^, systemic toxicity, and resistance^(10)^. Furthermore, vitreous seeding^(11)^ is also a major factor that leads to clinical failure of local or systemic treatments.

Recently, intraocular injection has been increasingly widely used in clinical treatment for RB vitreous seeding, as it successfully avoids complications of local radiotherapy^(7)^ and effectively breaks through the blood-retinal barrier. Intravitreal native CBP plus bevacizumab, which is under investigation, may be appropriate for controlling refractory RB seeds under the current investigation; however, it is ineffective for recurrent tumors^(12)^. In addition to concomitant intraocular injection showing a substantially increasing efficacy for saving eyes indicated for enucleation^(13)^, frequent intraocular injections will definitely increase the possibility of endophthalmitis and vitreous hemorrhage^(14)^. Therefore, a new drug delivery system offering greater efficiency must be developed to increase the efficacy and reduce the side effects of intravitreal CBP injection.

Nanoparticles is a newly emerging drug delivery tech nology. It can greatly enhance and prolong drug retention^(15)^. Via vitreous injection, it can penetrate through the blood-retina barrier efficiently. Biodegradable polymers of natural (albumin, chitosan, gelatin, and alginate) or synthetic {poly(lactic acid), poly(D, L-glycolide), PLAG^(16)^, poly(cyanoacrylate)} origin have been widely used as nano-carriers for ocular delivery^(17)^. The PLGA polymer, whose application in humans has been approved by the United States Food and Drug Administration, has been widely used in the preparation of spectacular nanoparticles (NPs) as drug delivery vehicles^(18)^. The ability of PLGA NPs to sequester plasmids, their nontoxic and safety characteristics, and rapid internalization enables gene transfer and expression in RPE cells^(19)^. These findings may be of potential use in designing future therapy strategies for ocular diseases of the posterior segment^(19)^. In addition, PLGA provides high encapsulation of active agents and exerts prolonged delivery and residence time, thereby minimizing the number and frequency of administrations^(20)^.

In in vitro experiments, because cell internalization occurred via clathrin-mediated endocytosis, surface-modified NPs achieved enhanced association and efficacy in RB cells relative to unmodified NPs^(16)^. Surface-modified PLGA has also been prepared using a copolymer to generate a NP formulation with enhanced long-circulating features^(15,21-23)^. Its physicochemical and biological properties such as water solubility, low toxicity, and anti-protein adsorption or cell adhesion have been improved to minimize the activation of immune systems^(24)^. Surface modification has been observed to improve NP transport from the anterior to the posterior segment of the eye and provide increased intravitreal NP stability^(16)^.

One strategy to limit side effects and prolong therapeutic efficacy is encapsulation of CBP in nanoparticles (NP). We modified the surface of PLGA using SA to optimize the ophthalmic delivery of CBP for the treatment of RB because in in vivo experiments, surface-modified PLGA increased the frequency of noninvasive topical eye drop administration to mouse retinal segments and greatly improved the delivery efficiency to the retina^(14)^, which indicates that overcoming the risk of invasive intraocular injection is no longer necessary in RB treatment. In this study, CBP-loaded surface-modified PLGA nanoparticles, scan electron microscopy (SEM), and Mastersizer 3000E laser particle size analysis was prepared. Enzyme-linked immunosorbent assay (ELISA) was performed to analyze the new delivery system. In addition, intracellular uptake, cell viability, and the proliferative activity assay of CBP-loaded nanoparticles were analyzed on a RB cell line (Y79) and compared with those of native CBP and void nanoparticles.

## METHODS

### 1. Materials

Sodium alginate and PLGA were purchased from Dae Jung (Korea). CBP was provided by Spectrum Pharmaceuticals. Fluorescein isothiocyanate (FITC), fetal bovine serum, bovine serum albumin, and acetic acid were obtained from Invitrogen (Leiden, The Netherlands). Propidium iodide was obtained from Bio-Rad (Hercules, CA, USA). The other reagents used (and vendors) were as follows: dimethyl sulfoxide (Welgene, Gyeongsangbuk-do, Korea), acetonitrile (HyClone, Logan, UT, USA), methyl thiazolyl tetrazolium (MTT) kit, and Ultrapure water (Milipore, Bedford, MA).

### 2. Preparation of PLGA/SA-PLGA nanoparticles

**Figure 1:**
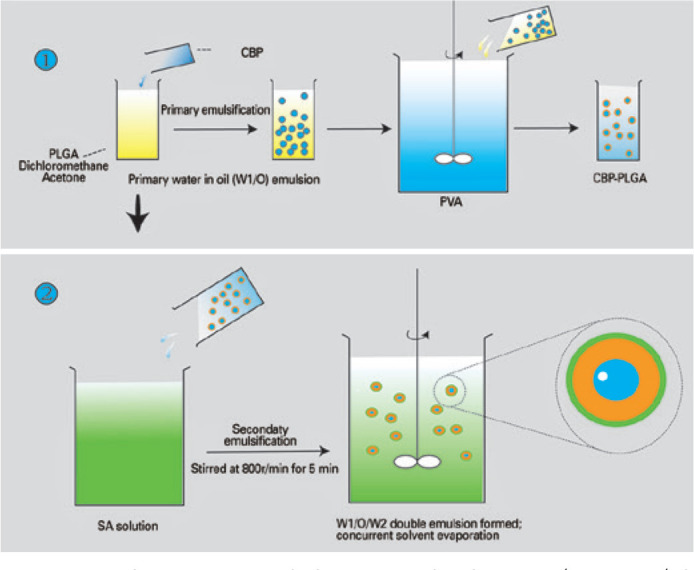
Schematic of the fabrication process using an adapted double-emulsion technique.

CBP-loaded PLGA ultrasound microbubbles were prepared using a double-emulsion technique as shown in figure 1. CBP-loaded SA-PLGA ultrasound microbubbles were also prepared using a double-emulsion technique as previously described^(25)^. Briefly, 15-µg CBP was mixed with 25-mg PLGA/SA-PLGA. Unloaded PLGA/ SA-PLGA was made simultaneously as the void group (Figure 1).

CBP= carboplatin; PLGA= poly(lactide-*co*-glycolide); W1/O= water/oil; PVA= polyvinyl alcohol; SA= sodium alginate; W1/O/W2= water/oil/water.

### 3. Morphological observation and particle size detection of CBP-loaded PLGA/SA-PLGA

#### 3.1. SEM studies

Particle morphology was examined using SEM (ZEISS). Images were captured, and results were analyzed with the Soft Imaging Viewer software.

The mean size and zeta potential of CBP-loaded PLGA/SA-PLGA microbubbles were analyzed using Mastersizer 3000E laser particle size analysis.

### 4. Encapsulation efficiency and loading capacity

The fabricated nanoparticle suspension was centrifuged at 18,000 rpm and 4°C for 50 min. The amount of CBP was calculated as the difference between the total amount of nanoparticles and the residual amount in the supernatant. The encapsulation efficiency (EE) and loading capacity (LC) of the nanoparticles were determined in triplicate and calculated using ELISA:

EE = [(Total amount of CBP-free CBP/Total amount of CBP]×100%

Drug LC = [(Total amount of loaded CBP/Total weight of nanoparticles)×100%

### 5. In vitro release studies

In vitro release studies of nanoparticles were performed using dialysis membrane (Sigma, USA). We dissolved 10 mg of CBP-loaded PLGA/SA-PLGA in 1 ml of phosphate buffer saline (PBS). The solution was sealed with the dialysis membrane. The whole system was placed in a 250-ml beaker containing PBS buffer and dialyzed with a constant temperature vibrator at 37°C ± 0.5°C and 100 rpm. At predetermined periods, 1 ml of the medium was extracted, and the cumulative CBP release percentage was analyzed using ELISA.

### 6. Cell culture

The human RB cell line Y79 was provided by ATCC (Manassas, VA, USA) and cultured in RPMI (Roswell Park Memorial Institute) 1640 medium with 5% fetal bovine serum (Sigma) and 1% streptomycin-pe nicillin (Invitrogen) at 37°C in a humidified 95% air/5% CO_2_ atmosphere.

### 7. Intracellular uptake

Y79 cells in the experimental groups were treated with 5-ml RPMI 1640 medium with 10% fetal bovine containing 50- µl CBP marked with 5′-FITC loaded with PLGA or SA-PLGA (0.25 µg/ul). In the control group, Y79 cells were treated with RPMI 1640 medium with 10% fetal bovine containing 50-µl CBP (0.25 µg/µl) marked with 5′-FITC. The two groups were cultured for a further 1-7 days. Fluorescent images were captured on confocal fluorescent microscopy at the time points.

### 8. Cell viability and proliferative activity assay

The cell viability and proliferative activity of the Y79 cells in the different groups were determined using MTT assay (Beyotime Company, Shanghai, China) and proliferating cell nuclear antigen (PCNA) with western blot (WB) after CBP treatment.

#### 8.1. MTT assay

The mixture was seeded into 96-well plates at a density of 5000 cells/well. We grouped the cells as follows: (1) control group; (2) unloaded PLGA: void group; and (3) (tenfold increase in concentration of 0.0005-5.000 µg/ml) native CBP or CBP-loaded PLGA/SA-PLGA. The Y79 cells were treated with these mixtures for 7 days. The cells were washed twice with PBS (HyClone, USA). A total of 25 µl of MTT (50 mg/ml) was added to each well, and the cells were incubated at 37°C for 4 h. Then, the culture medium of each well was replaced with 150 µl of dimethyl sulfoxide (Sigma) and shaken for 15 min. The next experiments were performed with 0.05 µg/ml CBP at 3, 5, and 7 days. Absorbance was measured at 490 nm using a microplate reader (Thermo).

#### 8.2. Western-blot assay

We grouped the Y79 cells as follows: (1) control group; (2) unloaded PLGA/SA-PLGA: void group; (3) 0.05 µg/ml native CBP; and (4) 0.05 µg/ml CBP-loaded PLGA or SA-PLGA. The experiments were performed for 3, 5, and 7 days.

The monolayer cultures were collected with cell scrapers and then lysed with 100 µL of cell lysis buffer on ice for 30 min. The cell lysates were centrifuged, and supernatants were collected. Total protein was prepared from each group. The protein concentrations in the supernatants were aliquoted and maintained using the BCA method (Biocolor, Shanghai, China) for further experiments. A total of 50 µg of protein per sample was electrophoresed with 10% polyacrylamide gel electrophoresis and transferred to nitrocellulose membrane (Millipore, Billerica, MA). It was blocked with 5% skimmed milk for 1 h at room temperature, and the nitrocellulose membrane was treated with primary antibodies (Abcam, Cambridge) at 4°C. After washing, the membrane was incubated with secondary antibodies (PCNA). The membrane was immersed in enhanced chemiluminescence solution and then exposed to an X-ray film. After the hybridization of the secondary antibodies, the resulting images were analyzed using ChemiImager 4000 (Alpha Innotech Corporation, California, USA).

### 9. Image acquisition and statistical analysis

The SPSS 13.0 statistical software was used to perform all the statistical analyses. After the treatment of the Y79 cells with different CBP concentrations, results of the overall comparison of the ratio value of MTT and protein expressions with those in the control group were analyzed using one-way analysis of variance, while the difference between the groups were compared using a Tukey honestly significant difference test. Differences with p values <0.05 were considered statistically significant.

## RESULTS

### Physicochemical characterization

**Figure 2:**
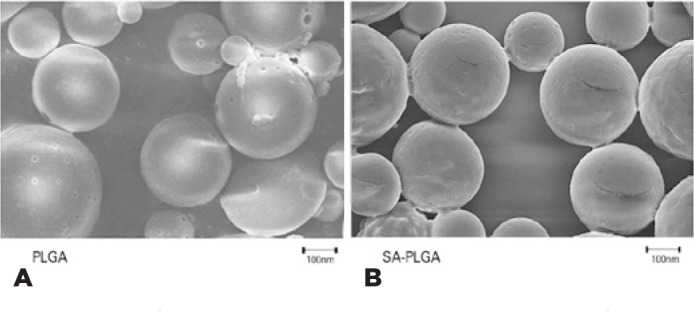
Scan electron microscopy micro-image of nanoparticles.

**Figure 3:**
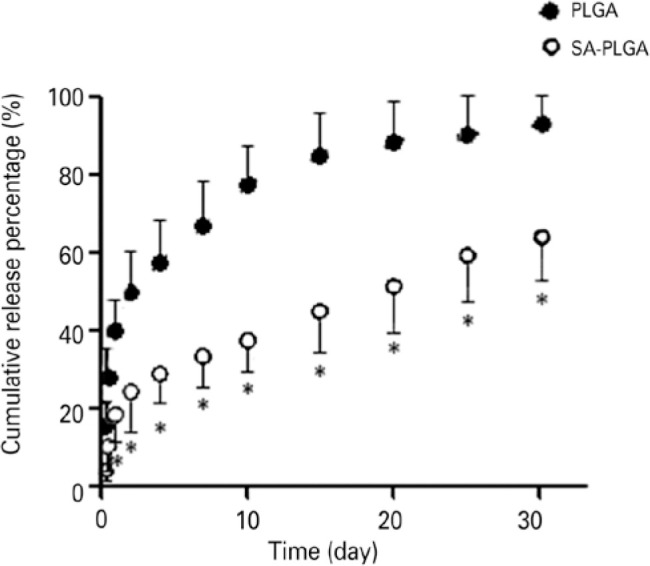
Carboplatin (CBP) nanoparticle release curve in vitro.In vitro release profile of CBP from nanoparticles in 0.01 M phosphate buffer saline, pH 7.4 at 37°C.

PLGA and SA-PLGA were round and smooth in shape. No adherence and rupture were observed in most SEM fields (Figure 2). The zeta potential of CBP-loaded PLGA ranged from -22.15 to -28.21 mV, and the mean was -(26.1 ± 3.1) mV. The zeta potential of CBP-loaded SA-PLGA ranged from -51.4 to -34.2 mV, and the mean was -(43.1 ± 8.1) mV. The particle sizes, EE, and LC of the nanoparticles are presented in table 1. The phenomenon of burst release was observed at 24 hours, and the release percentages of PLGA and SA-PLGA were 40.01% ± 8.2% and 18.86% ± 4.3%, respectively. Subsequently, slow release was observed, and the release percentage of PLGA and SA-PLGA reached 93.52% ± 5.8% and 63.12% ± 9.7% on day 30, respectively. The release curve is shown in figure 3.

**Table 1:** Particle size, encapsulation efficiency, and drug loading of different nanoparticle micro-bubble batches

		PLGA			SA/PLGA	
**Batch**	**Size (nm)**	**EE (%)**	**LC (CBP -**µ**g/mg)**	**Size (nm)**	**EE (%)**	**LC (CBP -**µ**g/mg)**
1	420.4	71.61	0.334	331.5	87.65	0.361
2	341.2	51.86	0.284	184.3	61.18	0.241
3	312.1	81.53	0.485	268.1	41.51	0.512
4	274.9	64.62	0.354	241.9	71.78	0.310
5	245.8	74.81	0.426	210.5	61.52	0.461
6	218.7	48.31	0.351	362.8	49.61	0.321
7	412.5	52.91	0.341	176.3	41.81	0.412
8	231.5	72.89	0.294	256.1	89.12	0.319
Mean ± SD	307.14 ± 95.43	64.82 ±12.38	0.359 ± 0.067	253.94 ± 66.53	63.02 ± 18.76	0.367 ± 0.089

The results of the experiment performed in triplicate are presented as mean ± SD, representing the results of three different experiments. CBP-loaded sodium alginate-poly(lactide-*co*-glycolide) (SA-PLGA) is released more slowly than PLGA (*p<0.05).

### Intracellular uptake

**Figure 4:**
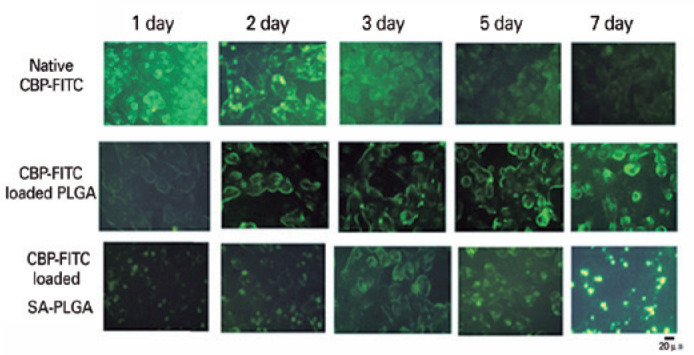
Time course study of intracellular retention of fluorescencelabeled carboplatin (CBP) in Y79 cells. Cells were treated with native CBP-fluorescein isothiocyanate (FITC), CBP-FITC-loaded poly(lactide-*co*-glycolide) (PLGA), and sodium alginate-PLGA (SA-PLGA) in growth medium. A decrease in green fluorescence intensity with native CBP-FITC incubation can be observed in the cells, whereas increased fluorescence with CBP-FITC-loaded PLGA and SA-PLGA incubation lasted up to day 7. Furthermore, CBP-FITC released from SA-PLGA in cytoplasm appeared more condensed than native CBP and PLGA.

The positive intracellular uptake of SA-PLGA or PLGA was extremely high as compared with that of native CBP-FITC (Figure 4).

### Cell viability assay

**Figure 5:**
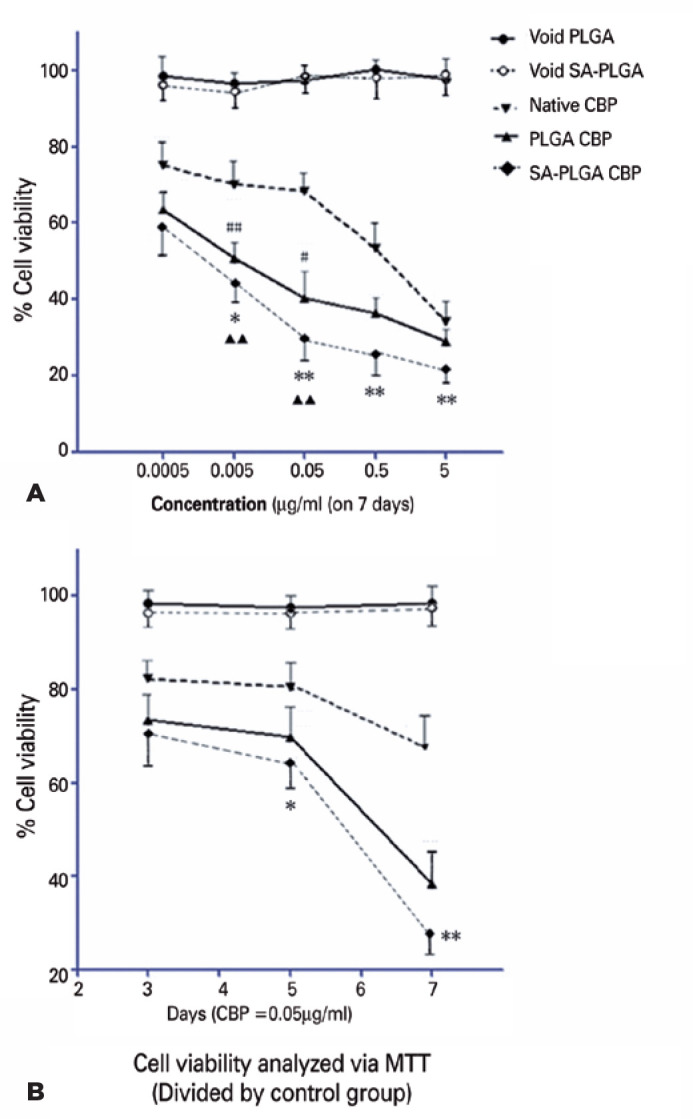
Decreased cell viability in RB Y79 in the native carboplatin (CBP) group. Both poly(lactide-*co*-glycolide) (PLGA) and sodium alginate-poly(lactide-*co*-glycolide) (SA-PLGA) promoted anti-metabolism in RB Y79. Y79 cell viability was measured using a MTT assay. CBP-loaded nanoparticles showed a greater inhibitory effect on Y79 than did native CBP at all concentrations or time points (p<0.05). Compared with CBP-loaded PLGA, CBP-loaded SA-PLGA exhibited a higher inhibitory effect at all concentrations or time points (*p<0.05, **p<0.001; Figure 5).

No significant difference in inhibition of Y79 viability was found between void PLGA and void SA-PLGA. The cell viability of the void groups divided by the control group was nearly 100%, which also demonstrated that no cytotoxicity was detected in the void nanoparticle groups. The doseand time-dependent cytotoxicities of native CBP and CBP-loaded nanoparticles were measured using the MTT assay in the Y79 cells. The results demonstrated that the viability of the Y79 cells was downregulated by the different gradient concentrations of native CBP, CBP-loaded PLGA, and SA-PLGA as compared with that in the void group (p<0.01; Figure 5A). The stronger anti-metabolism activities of CBP-loaded PLGA or SA-PLGA were observed to be dose dependent as compared with those of the native drug on day 7 (p<0.05; Figure 5A). On day 7, 0.005 µg/ml CBP-loaded PLGA or SA-PLGA showed a greater inhibitory effect than that of 0.05 µg/ml native CBP. Furthermore, 0.05 µg/ml CBP-loaded PLGA or SA-PLGA also exhibited a greater inhibitory effect than that of 0.5 µg/ml native CBP (^##^p<0.001, ^#^p<0.05, ^▲▲^p<0.001; Figure 5A), which indicated that a lower dosage of CBP-loaded nanoparticles could attain a greater inhibitory effect than that of native CBP. Compared with PLGA, CBP-loaded SA-PLGA showed a stronger inhibitory effect at concentrations ≥0.005 µg/ml on day 7 (*p<0.05, **p<0.001; Figure 5A).

We also observed that the inhibitory effect of native CBP (concentration, 0.05 µg/ml) was weaker than that of CBP-loaded nanoparticles (p<0.01; Figure 5B). In addition, the inhibitory effect of native CBP did not increase significantly from days 3 to 5 (p>0.05; Figure 5B). Significant differences were found between the native drug and CBP-loaded nanoparticles during 1-7 days. On day 7, an even stronger anti-metabolism effect was detected (38% inhibition by CBP-loaded PLGA and 27.5% by loaded SA-PLGA vs 67% by native CBP; p<0.05; Figure 5B). We found no significant difference between CBP-loaded PLGA and loaded SA-PLGA on day 3 (p>0.05; Figure 5B). However, the inhibitory effect of CBP-loaded SA-PLGA was more significant than that of CBP-loaded PLGA as time passed (*p<0.05, **p<0.001; Figure 5B).

### Cell proliferation assay

**Figure 6:**
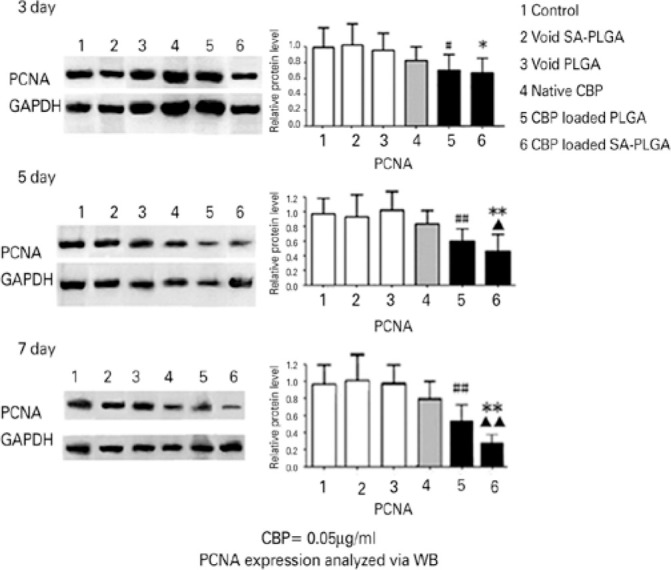
Cell proliferation was analyzed using western blot. After 3, 5, and 7 days, the inhibitory effect of native carboplatin (CBP) did not increase over time (p>0.05). However, CBP-loaded sodium alginate-poly(lactide-*co*-glycolide) (SA-PLGA) exhibited a much stronger inhibitory effect on proliferating cell nuclear antigen (PCNA) expression than did native CBP and CBP-loaded PLGA on day 7 (p<0.01).

Time-dependent anti-proliferation was measured using a WB assay. No significant differences were found among the control, void PLGA, and void SA-PLGA groups (p>0.05), which demonstrated no cytotoxicity of void nanoparticles in Y79 cells. Native CBP did inhibit the PCNA expression at all the time points (p<0.05). However, over time, the inhibitory effect did not increase (p>0.05). CBP-loaded PLGA or SA-PLGA showed a higher inhibitory effect on the PCNA expression than difcdvvd native CBP (^#^p<0.05; ^##^p<0.01; *p<0.05; **p<0.01) and the void nanoparticles (p<0.01). On day 5, CBP-loaded SA-PLGA showed a stronger inhibitory effect on PCNA expression than did CBP-loaded PLGA (^▲^p<0.05). Moreover, on day 7, the difference between SA-PLGA and PLGA was more significant (^▲▲^p<0.01; Figure 6).

GAPDH: glyceraldehyde-3-phosphate dehydrogenase.

## DISCUSSION

Carboplatin is a cytotoxic and second-generation platinum-based anti-neoplastic agent that is a better substitute for cisplatin in combination regimens, as its dose can be tailored to the patient’s renal function and its non-hematological toxicity profile is more favorable^(26)^. It is frequently used in chemotherapeutic treatments for RB children^(27)^. The anti-neoplastic mechanism of CBP is its interaction with DNA, whereby its Pt(NH_3_)_2_ moiety binds covalently to two adjacent guanine bases. These Pt-DNA adducts are believed to lead to the eventual death of cancer cells^(26)^. CBP is highly efficacious against numerous neoplasms, but has defects that are related to its moderate leukemogenic potential and toxicity to normal cells^(28)^. Thus, systemic administration of CBP for RB treatments could cause many side effects in children. Although intravitreal injection of CBP can break through the blood-retinal barrier with improved local drug concentrations, the injection has many complications, of which endophthalmitis and vitreous hemorrhage are the most severe and can lead to irrevocable visual loss.

As endophthalmitis and vitreous hemorrhage can be induced by frequent intravitreal injection^(14)^, a drug delivery system with lower risks of complications and higher efficacy is warranted for chemotherapy against RB. CBP-loaded PLGA or SA-PLGA were used in this research to intervene the metabolism of Y79 cells. The standard deviation of SA-PLGA particle size was smaller than that of PLGA particles, which demonstrates that the surface modification with SA could improve the homogeneity of nanoparticles. The zeta potential of PLGA modified by SA was higher than that of PLGA. The higher zeta potential of the nanoparticles indicated more electrokinetic stability of the formulation, as nanoparticles with a zeta potential >30 mV were more stable owing to the stronger repulsive forces among the particles preventing aggregation^(29)^. More stable physicochemical characterization of SA-PLGA also means better performance in the following clinical uses: In the CBP release curve, CBP-loaded SA-PLGA was released more slowly than PLGA. The burst release percentage of SA-PLGA was 18.86% ± 4.3% and was much lower than that of PLGA, which means that SA-PLGA prolonged drug release more effectively. The slow release of CBP-loaded SA-PLGA showed a promising prospect for reducing the frequency of intravitreous injection, which might lead to various risks in RB children. Furthermore, in an intracellular uptake experiment, the uptake was increased gradually by both SA-PLGA and PLGA over time as compared with the decreased green fluorescence intensity with native CBP-FITC incubation. In addition, CBP-FITC-loaded SA-PLGA were better absorbed in the cells, and the fluorescence intensities were more condensed within the cytoplasm than the cell membrane fluorescence staining with CBP-loaded PLGA. Several possibilities might explain the mechanisms of the cellular uptake of SA-PLGA. One possibility is that SA on the surfaces of PLGA may increase cell membrane fluidity, which leads to endocytosis activation. Therefore, we considered that SA might be a candidate PLGA surface modifier for use in a cellular drug delivery system.

The inhibitory effect of CBP-loaded nanoparticles against the Y79 cell line was evaluated using the MTT assay and western blot as follows: although native CBP inhibited the viability and proliferation of Y79 cells, CBP nanoparticles exhibited a more powerful inhibitory effect on the cells than did native CBP. Furthermore, CBP-loaded SA-PLGA showed a much stronger inhibitory effect than did CBP-loaded PLGA on day 5. On day 7, even a lower concentration of SA-PLGA or PLGA exhibited a much stronger inhibitory effect on cell viability than did native CBP, which indicated that a lower dosage could be used to avoid side effects without losing its effectiveness. SA-PLGA with stronger inhibition on the Y79 cell line could be interpreted by its higher efficacy on endocytosis, which was observed in the intracellular uptake experiment. In addition, owing to the high penetration of surface-modified PLGA into eye tissue using topical eye drop instillation in vivo^(14)^, SA-PLGA is a much safer drug delivery system than vitreous CBP injection in children with RB.

Although CBP-loaded SA-PLGA showed a remarkable suppression of the proliferation and viability of Y79, still other issues must be managed. Owing to corneal endothelial cell non-regeneration, drugs that interfere with cell metabolism must be safe and nontoxic. On account of the CBP-loaded SA-PLGA characteristics of slow release, its cytotoxicity to corneal endothelial cells would be long-term and irreversible. Therefore, cytotoxicity to corneal endothelial cells must be further detected. Furthermore, how intraocular liquid circulation such as the aqueous humor influences the effects of CBP-loaded SA-PLGA should be researched in vivo experiments in the future.

In our future research, the use of SA-PLGA eye drops in vivo experiments must be examined. The formula that offers the best zeta potential for the eye surface, stabilization of compounds, and prevention of the aggregation or sedimentation must be developed. Additional research should focus on intravitreal CBP concentration determination, pharmacokinetics, and the combination with other transfection vectors such as lipidosome to enhance uptake efficacy. Security for human eye applications is warranted in the proposed objectives for future research.

In conclusion, CBP-loaded SA-modified PLGA exhibited a much stronger and prolonged inhibitory effect than did PLGA and native CBP. It shows great therapeutic prospect in the treatment of RB. Our results suggest that the developed formulation may improve the targeted therapy for malignant eye tumors in the future and supersede previous invasive therapies.
